# Lessons Learned From Applications of the Stage Model of Self-Regulated Behavioral Change: A Review

**DOI:** 10.3389/fpsyg.2019.01091

**Published:** 2019-05-09

**Authors:** Ellis Keller, Charis Eisen, Daniel Hanss

**Affiliations:** Department of Social Sciences, Hochschule Darmstadt – University of Applied Sciences, Darmstadt, Germany

**Keywords:** stage models, pro-environmental behavior, behavioral change, goal-directed behavior, self-regulation, tailored information, intervention, sustainable development

## Abstract

Stage models are becoming increasingly popular in explaining change from current behavior to more environmentally friendly alternatives. We review empirical applications of a recently introduced model, the stage model of self-regulated behavioral change (SSBC). In the SSBC, change toward pro-environmental behavior takes place in four, qualitatively different stages (predecisional, preactional, actional, and postactional) which are each influenced by constructs taken from theories previously established to describe and predict pro-environmental behavior. We performed a systematic literature search to retrieve peer-reviewed SSBC-based studies. The review includes 10 studies published between 2013 and 2018, six of which employed a cross-sectional, three an interventional and one a correlational longitudinal design. The cross-sectional and longitudinal studies generally support the model, although there are some irregularities that warrant further investigation. The interventional studies found stage-tailored informational measures to be more effective than non-stage-tailored measures in promoting stage progression and behavioral change. Furthermore, we identified several challenges that researchers may face when applying the SSBC. These include whether and how to analyze multiple behavioral alternatives; how to address the challenge of measuring a comprehensive model while keeping questionnaire length manageable; selecting and defining the role of model constructs in a behavioral context while keeping results comparable; and establishing a validated and reliable tool to diagnose a person’s stage of change. Based on these insights, we develop recommendations for researchers designing SSBC studies, in order to support a founded and efficient advancement of the theory which will then serve both researchers and practitioners aiming to promote pro-environmental behavior.

## Introduction

Transitioning to environmentally friendly behavior can be a complex process that involves many different influencing factors and often takes place not at one point in time, but as a series of events and tasks. Environmental psychology has increasingly conceptualized and implemented stage models to reflect this perspective. One of these, the *stage model of self-regulated behavioral change* (SSBC, Bamberg, 2013b), combines the *model of action phases* (MAP; Gollwitzer, 1990) andstatic theories established in environmental psychology (e.g., the norm activation model, detailed below) to create a comprehensive framework for explaining behavioral change. Thereby, it aims to extend previous stage models by cumulatively considering both the dynamic, longitudinal processes reflecting multiple stages of decision making, as well as the factors influencing each of those single decisions or stage transitions.

**FIGURE 1:**
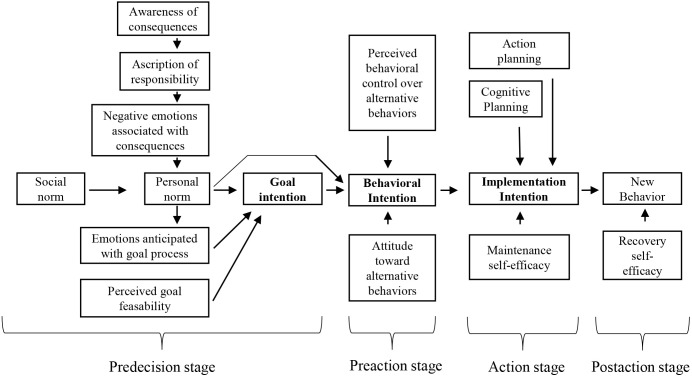
The stage model of self-regulated behavioral change, adapted from Bamberg (2031b).

In the SSBC, behavioral change is modeled as a series of stages, marked by different tasks and by different intentions that indicate the passing to the next stage (seen in Figure 1).

In the first stage, *predecision*, the current behavior needs to be perceived as problematic, leading to an intention to reduce this behavior or negative consequences (*goal intention*). Variables assumed to influence this type of intention are based on the *norm activation model* (NAM; Schwartz and Howard, 1981) as displayed in Figure 1. In the next stage, *preaction*, an individual chooses an alternative behavior to achieve the goal of reducing his/her current behavior or negative impact. The intention to perform this newly chosen behavior is termed *behavioral intention* and is influenced by variables of the *theory of planned behavior* (TPB; Ajzen, 1991). Next, the new behavior has to be implemented in everyday life, determined by the strength of a person’s *implementation intention* which is in turn influenced by planning abilities and maintenance self-efficacy. Finally, in the *postaction stage,* the new behavior has to be maintained without permanent relapse.

Since its introduction, the SSBC has been applied to a variety of contexts in a number of different research designs. Especially for newly conceptualized models, it is vital to track the success of their application to be able to gauge necessary changes or potentially useful applications. This is particularly important in the field of sustainability and environmental psychology which naturally targets pressing issues. To facilitate further development and application of the model, this review aims to summarize the research around the SSBC and to provide insights into its success, but also its limiting factors.

## Materials and Methods

We selected articles according to the following criteria: (1) Studies had to be based on the SSBC, meaning they had to (1a) include multiple SSBC key constructs (in a correlational study) or use the SSBC as an explicit framework (in an intervention study), and (1b) be published since 2013, i.e., after the introduction of the model, (2) apply the model to an environmentally relevant behavior, (3) report original research, and (4) be published in English or German language.

Titles were searched in a four-step search strategy: Firstly, we searched for articles that had cited the initial publication of the SSBC (Bamberg, 2013b) in Web of Science (*n* = 62) and Google Scholar (*n* = 147). Our reasoning was that the SSBC was introduced recently enough to most likely have been cited by all authors applying the model. As a second step, we applied the search terms (stage model^∗^ OR stage based) AND (behavior OR behavior^∗^ change) AND (environment^∗^ OR sustainable^∗^) to Web of Science (*n* = 2748) and PsycINFO (*n* = 93), limited to titles published since 2013 and to articles only. These searches were last conducted on February 22nd 2019. As a third step, we contacted the author of the theory to learn about any additional studies (*n* = 7). After deleting duplicates (*n* = 73), this led to a total of 2982 studies.

We then screened the abstracts and titles applying the eligibility criteria presented above, which resulted in 21 potentially relevant articles. When screening the full texts, five were excluded because they only applied a very limited number of SSBC concepts (e.g., a TPB study including a “norms” variable), two were excluded because they applied stage models other than SSBC, and four were excluded because they had used the SSBC for the conception of their study or intervention, however, without evaluating the model. In total, 10 articles were identified as relevant for the review.

As a fourth step, we hand searched the reference lists of the selected studies, as well as prominent journals in the field of environmental psychology. This resulted in no additional studies.

## Results

Table 1 displays a summary of the studies included in the review. In the following, we will refer to the studies as numbered in Table 1.

**Table 1:** Summary of articles included in review.

No.	Author(s)	Type of study	Sample size	Behavioral domain	Type of decision	Key findings
1	Bamberg, 2013b	Correlational (cross-section)	*N* = 908	Car use to public transport and cycling	Low-cost, repeated decision	Structural equation modeling (SEM) path and measurement model with acceptable model fit. Stages thresholds predicted by model constructs. Model constructs differ between stages (no pairwise comparisons).
2	Klöckner, 2017	Correlational (cross-section)	*N*_1_ = 746 *N*_2_ = 2967	Beef consumption to reducing portion sizes, replacing with other meats or fish, or eating vegetarian	Low-cost, repeated decision	SEM path model with acceptable fit. Model constructs differ between stages according to model predictions in behavior and goal intention, less clear in behavioral intentions and implementation intentions.
3	Schaffner et al., 2017	Correlational (cross-section)	*N* = 1818	Moving into an energy-efficient home	High-cost, investment decision	Multi-group model (predecision and preaction constructs predicting behavioral intentions) with acceptable fit. Stage predictors largely differ between stages according to model predictions.
4	Ohnmacht et al., 2018	Correlational (cross-section)	*N* = 1818	Postponing replacement smartphone buys	Low- to high-cost decision	Stages membership predicted by model constructs, independent from socio-demographic variables. Model constructs differ between stages (no pairwise comparisons).
5	Olsson et al., 2018	Correlational (cross-section)	*N* = 794	Car use to public transport, bike, walk, or carpool	Low-cost, repeated decision	Stages membership predicted by model constructs, independent from socio-demographic variables.
6	Weibel et al., 2018	Correlational (cross-section)	*N* = 1818	Reducing meat consumption	Low-cost, repeated decision	Stages membership predicted from model constructs, but include irregularities. Stage membership was also predicted from socio-demographic variables. Model constructs differ between stages (no pairwise comparisons).
7	Klöckner, 2014	Correlational (longitudinal, questionnaire every second day over 2 months)	*N* = 113	Purchasing an e-car	High-cost, investment decision	Stage transitions generally followed pattern predicted by SSBC (both forward and backward). Intentions partially linkable to transitions. Intentions mostly predicted by model predictors.
8	Bamberg, 2013a	Intervention study using stage-tailored phone campaign	*N* = 291	Car use to public transport, cycling or walking	Low-cost, repeated decision	Stage-tailored campaign was more influential on behavior and stage progression than standardized information and no information.
9	Klöckner and Ofstad, 2017	Intervention study using stage-tailored websites	*N*_1_ = 389 *N*_2_ = 869 *N*_3_ = 3508	Beef consumption to reducing portion sizes, replacing with other meats or fish, or eating vegetarian	Low-cost, repeated decision	Tailored-information website outperforms randomly tailored website, full website, and no intervention on stage progression and behavior.
10	Sunio et al., 2018	Intervention study using website and smartphone app for participants to self-tailor after recommendations	*N* = 241	Car use to environmentally friendly modes of transport for University commute	Low-cost, repeated decision	Intervention had effect on behavior and later stages of change (compared to no-intervention control group). SEM path model had to be significantly adapted to achieve adequate fit.


Six studies applied the SSBC in a cross-sectional design (1–6), one in a longitudinal correlational design (7) and three in an intervention design (8–10). The type of behaviors under study (see Table 1 for description and categorization of behaviors) can largely be placed into two categories: seven studies analyzed low-cost, repeated decisions (1–2, 5–6, 8–10) and two studies analyzed high-cost, investment decisions (3, 7), with one study addressing a behavior that falls between the two categories (4).

Authors have pursued different approaches in analyzing various components of the model. In the following, we will first summarize the main findings of the correlational and interventional studies, followed by a look at key challenges when applying the SSBC.

### Results From Cross-Sectional Studies

In general, group comparisons between stages of change were found to be significant and meaningful. Model constructs and behaviors were found to differ in intensity between stages (1, 4, and 6), and for those studies that conducted pairwise analyses on these differences (2, 3), the patterns were largely as predicted by the model (e.g., current behavior should significantly decrease in the postactional stage, when an individual has adopted an alternative behavior). However, there are some irregularities that suggest that stages cannot always be separated as easily as modeled, especially in the preactional and actional stages. This might, however, be partly caused by the studies’ cross-sectional representation of a process-oriented model.

Structurally, the SSBC was supported by three studies using structural equation modeling or multi-group modeling (1–3), although relationships differed in significance and effect sizes both between and within the studies’ investigated behaviors. A cross-sectional path model estimated as part of an interventional study (10), however, had to be significantly modified in choice of constructs and paths to result in an adequate fit, providing only limited support for the SSBC.

Additionally, logit regression models were used by four authors (1, 4–6) to predict the stages of change from model constructs. While these, too, had some irregularities in which constructs were associated with various non-predicted stages, they generally supported model assumptions, with model constructs mostly outperforming socio-demographic variables in their predictive power.

### Results From Interventional Studies

Three studies designed and evaluated stage-tailored information campaigns based on the SSBC (8–10). In all three studies, authors based the content of their information modules on respective stage tasks and factors predicted to influence the respective intention. Two (9–10) designed a web-based tool that participants accessed after self-diagnosing their stage, either with restricted stage-tailored access or with prior recommendations as to which website modules would be most useful for a person’s stage. One (8) designed a phone-based intervention campaign complemented by tailored information packages.

Approaches in evaluating the interventions differed (see Table 1), but tailored information always significantly influenced stage progression and behavior, and outperformed no-information or non-tailored information conditions. However, only one study (9) included a control group to assess whether there was a genuine effect of the tailoring of the information as opposed to an effect of the reduced amount of information, or even simply the presence of information. While the stage-tailored information in this study did outperform a mismatched-tailored-information condition, more experimental research is needed to provide further evidence on the exact role and effectiveness of stage-tailored information.

### Results From Longitudinal Study

Only one study (7) conducted a longitudinal, non-interventional investigation, with questionnaires administered every 2 days for a period of 2 months. Stage transitions mostly followed predicted patterns and were partially associated with the respective intentions. These results provide considerable support for the model, successfully linking the process of behavior change to longitudinal data.

### Challenges in the Application of the SSBC

#### Modeling (Multiple) Alternative Behaviors

As the SSBC describes behavioral change from a current behavior to a new, more environmentally friendly behavior, there are several decisions that authors have to make when choosing a study design. One central decision concerns whether it is the old or the new behavior that is the focus of the study, that is to say: Do we want to model behavioral change *away* from an old behavior or do we want to model change *toward* a new behavior – or both? There are advantages and disadvantages to each approach.

For example, three of the reviewed studies have taken the first approach of changing *away* from current behavior (5, 6, and 10). This means that in questionnaire items, no concrete alternative behaviors were mentioned. For example, in this approach a measure of behavioral intention would include a statement similar to “I intend to perform a behavior other than [old behavior].” This approach performs well in making sure all participants feel addressed, as they can choose their own alternative behavior. However, any investigation into which alternative behavior works best for whom is limited.

In a study design focusing on change *toward* a specific behavior (3, 4, and 7) participants respond to items only regarding this one alternative behavior (i.e., “I intend to perform behavior X”). Consequently, there is limited insight into possible alternative behaviors that might lead to a reduction of the old, problematic behavior, or into alternative, equally problematic behaviors that participants might adopt under wrong assumptions. However, this approach will serve well, if there is a behavior that is to be promoted for specific reasons. While information regarding other alternatives might be lost, questionnaires for this study design are shorter and might reduce recruiting efforts.

There are also efforts to model both change away from current and toward alternative behaviors, combining benefits of the two. For example, researchers can provide multiple behavioral alternatives to which participants respond (2, 8) or additionally let participants suggest an alternative behavior of their own, should none of the alternatives apply to them (1). While this results in longer questionnaires, it means that behavioral alternatives can be compared and the interpretation of results can be linked to specific behaviors.

#### Investigating Intentions

Within the SSBC, goal, behavioral, and implementation intentions act as the thresholds for stage progression. However, authors have taken different approaches to including these in study designs, making comparisons between their effectiveness somewhat difficult. Most studies included all intentions, but these then referred to either one alternative behavior (3, 4, and 7), to a non-specific alternative behavior (5, 6, and 10), or were operationalized for each of the proposed alternative behaviors (1, 2). One study (3) only measured behavioral intentions, which were then predicted by constructs of several stages. These considerations are related to the issue of how to model current and alternative behaviors (see above), as alternative behaviors are closely related to intentions, especially of the later stages. As the SSBC conceptualizes an increase in intentions as a signal for respective stage transitions, the relationship between intentions, stage transitions, and alternative behavior needs to be more closely examined, in correlational, longitudinal and interventional research alike.

#### Operationalization and Forced Choice of Variables

Due to the large number of factors, comprehensive item batteries with two to three items per construct can result in questionnaires that are of unreasonable length to administer – especially for studies with several measurement points. Authors have used single-item batteries (7, 10), and the majority have focused on the most important constructs or stages. However, since different behaviors often produce different results, even in similar behavioral domains (e.g., 2), it is difficult to decide *a priori* which constructs are less important.

#### Behavior and Stage Diagnosis

Another common issue, though not limited to the application of the SSBC, is the operationalization of behavior. Behavior, if measured at all, is self-reported and may therefore be biased. This bias can then extend to the validation of stage assignment, which is often cross-checked with behavior. Validating stage diagnosis measures is an essential step toward the validation of the SSBC. As described by the author of the theory: “There is some discrepancy between how the stages are theoretically conceptualized (i.e., in terms of tasks and mind sets) and how the measure operationalized stage membership (current behavior and motor car use goal)” (Bamberg, 2013b, p. 158).

## Conclusion

In summary, the SSBC has received support for its key constructs and has been successfully applied to different contexts and behaviors, including both high-cost and low-cost behaviors. Interventions based on the SSBC have proven to be more effective than non-tailored solutions and have indicated promising possibilities for systematic intervention designs. However, there are also irregularities found in the study results – for example, stage membership sometimes seems more ambiguous than assumed by the model. Additionally, results from applications to different behavioral domains and even different behavioral alternatives have shown that especially the explanatory power of model predictors can vary significantly. Consequently, it is essential to continue to apply the SSBC to different contexts to gain further insight into its validity. During this process, it is important that researchers consider the different approaches they can take with regard to study design and conceptualization, in order to ensure comparability and maximize effectiveness of possible interventions. Based on our observations outlined above, we recommend the following:

• In cross-sectional designs, consider whether and how alternative behaviors are selected and portrayed. Depending on the situation, multiple alternative behaviors may or may not exist– if they do, consider a study design that includes alternative behaviors to appeal to a maximum number of participants, while also considering comparability of alternative behaviors to improve possible recommendations for applications.
• With regard to the operationalization of variables, consider that instruments should be kept down to a manageable length without sacrificing reliability. If a selection of variables is necessary, make that selection carefully and in close relation to your research question.
• In an interventional design, researchers could gain more insight into mechanisms of behavior change by evaluating multiple model constructs, if possible. These insights would extend the information gained from investigating change of behavior and stage transition. For example, an estimation of how effective which tailored information was, and why, would benefit future intervention design.
• In an interventional design, include not only a no-information and all-information group, but also a control group that receives mismatched tailored information to control for the effect of reduced information.
• Substantial insight could be gained from more longitudinal studies with multiple points of measurement, be it for low-cost or high-cost decisions, or in a correlational or interventional design. As the SSBC is explicitly modeled as a process, actual longitudinal data could provide additional information on persons’ transitions to more environmentally friendly behavior.

While this list of recommendations is not exhaustive, it provides future research with a starting point when developing research designs. Researchers should also consider information to be gained from additional resources that could not be considered in this review, such as a review of smart meters in the framework of the SSBC (Nachreiner et al., 2015) or studies which have applied, though not evaluated interventions based on the SSBC to inspire their work (Bamberg et al., 2015; Mack and Tampe-Mai, 2016). The latter will also be relevant for practitioners looking to design theory-based interventions.

Last but not least, researchers should keep in mind that even though stage models such as the SSBC strive to reflect the dynamics of a decision-making process, they still represent this process in a linear, static fashion, simplifying complex interdependencies and feedback loops that can be assumed to take place in real-life decision making. Research should always consider whether a specific context necessitates additional consideration of these factors and, at the very least, acknowledge them in the specification or interpretation of any stage model.

## Author Contributions

EK performed the review and took the lead in writing the manuscript. All authors edited and contributed to the manuscript, and conceived of the present idea and developed the method.

## Conflict of Interest Statement

The authors declare that the research was conducted in the absence of any commercial or financial relationships that could be construed as a potential conflict of interest.
